# Botulinum toxin-induced acute anterior uveitis in a patient with Behçet’s disease under infliximab treatment: a case report

**DOI:** 10.1186/s13256-017-1288-1

**Published:** 2017-05-04

**Authors:** Hirofumi Sasajima, Syunsuke Yagi, Hiromu Osada, Masahiro Zako

**Affiliations:** 1 Department of Ophthalmology, Aichi Medical University, Nagakute, Aichi Japan; 2 Department of Ophthalmology, Asai Hospital, 178-1 Yakata-cho, Seto, Aichi 489-0866 Japan

**Keywords:** Behçet’s disease, Botulinum toxin, Endotoxin-induced uveitis, Infliximab, Lipopolysaccharide

## Abstract

**Background:**

Injections of lipopolysaccharide in animal models generate acute anterior uveitis (also known as endotoxin-induced uveitis), but the effects of lipopolysaccharide injection are unknown in humans. We describe an unusual case in which acute anterior uveitis was dramatically activated subsequent to botulinum toxin injection in a patient with Behçet’s disease but the acute anterior uveitis was satisfactorily attenuated by infliximab.

**Case presentation:**

A 53-year-old Japanese man had normal ocular findings at his regularly scheduled appointment. He had been diagnosed as having incomplete-type Behçet’s disease 11 years before. Three years after the diagnosis he was given systemic infusions of 5 mg/kg infliximab every 8 weeks and he had not experienced a uveitis attack for 8 years with no treatment other than infliximab. Two days after the eye examination, he received intracutaneous botulinum toxin injections to treat axillary hyperhidrosis on both sides. Three hours after the injections, he noted rapidly increasing floaters in his right eye. Four days after the injections, his right eye showed severe acute anterior uveitis with deteriorated aqueous flare and anterior vitreous opacity. He received his scheduled infliximab injection, and the right acute anterior uveitis immediately attenuated.

**Conclusions:**

Botulinum toxin may have clinical effects similar to those of lipopolysaccharide in endotoxin-induced uveitis models. To the best of our knowledge, this is the first report to suggest that botulinum toxin may trigger acute anterior uveitis, although the precise mechanism is still unclear.

## Background

In animal models, systemic and local injection of lipopolysaccharide (LPS) induces acute anterior uveitis (AAU), which is also known as endotoxin-induced uveitis (EIU). LPS directly affects the production of inflammatory mediators by cells in the anterior uvea, leading to EIU [1]. The inflammation associated with EIU is characterized by an acute breakdown of the blood–aqueous barrier (BAB) within 3 hours after LPS injection and the subsequent development of clinical disease [2].

LPSs are large molecules consisting of a lipid and a polysaccharide that includes O-antigen. They are found in the outer membrane of Gram-negative bacteria, implicating these bacteria in AAU pathogenesis. Kufoy *et al*. demonstrated that infection with Gram-positive bacteria might also cause uveitis, since rabbit and rat eyes are susceptible to muramyl dipeptide, which is found in the cells walls of both Gram-positive and Gram-negative bacteria [3]. Thus, it is possible that botulinum toxin, a neurotoxic protein produced by the Gram-positive anaerobic bacterium *Clostridium botulinum*, might also induce EIU.

EIU induced by LPS injection is characterized by increased expression of tumor necrosis factor alpha (TNF-α). TNF-α messenger ribonucleic acid (mRNA) peaks 3 hours after LPS injection [4]. Anti-TNF-α treatment attenuates LPS-induced EIU in animal models. Specifically, it reduced LPS-induced increases in leukocyte rolling, adhesion, and vascular leakage in a rat model of inflammatory uveitis [5]. These findings suggest that TNF-α is critically involved in the pathogenesis of EIU. Thus, anti-TNF-α therapy is an effective means of abrogating LPS-induced EIU in animal models.

Of interest, LPS induces excessive TNF-α-production by cells from patients with Behçet’s disease (BD). LPS-stimulated production of TNF-α is significantly increased in peripheral blood monocytes from patients with BD [6].

Recently, we saw a patient with BD who underwent subcutaneous injections of botulinum toxin to treat axillary hyperhidrosis. Shortly after, he developed AAU. This patient had been stable, with his BD under control for 8 years; during this time, he had undergone regularly scheduled infliximab treatment. When he underwent his next scheduled infliximab treatment after the event, the AAU improved to almost normal levels. To the best of our knowledge, this is the first report to describe an EIU-like condition associated with botulinum toxin injection and effective attenuation by infliximab in a patient with BD.

## Case presentation

**Fig. 1:**
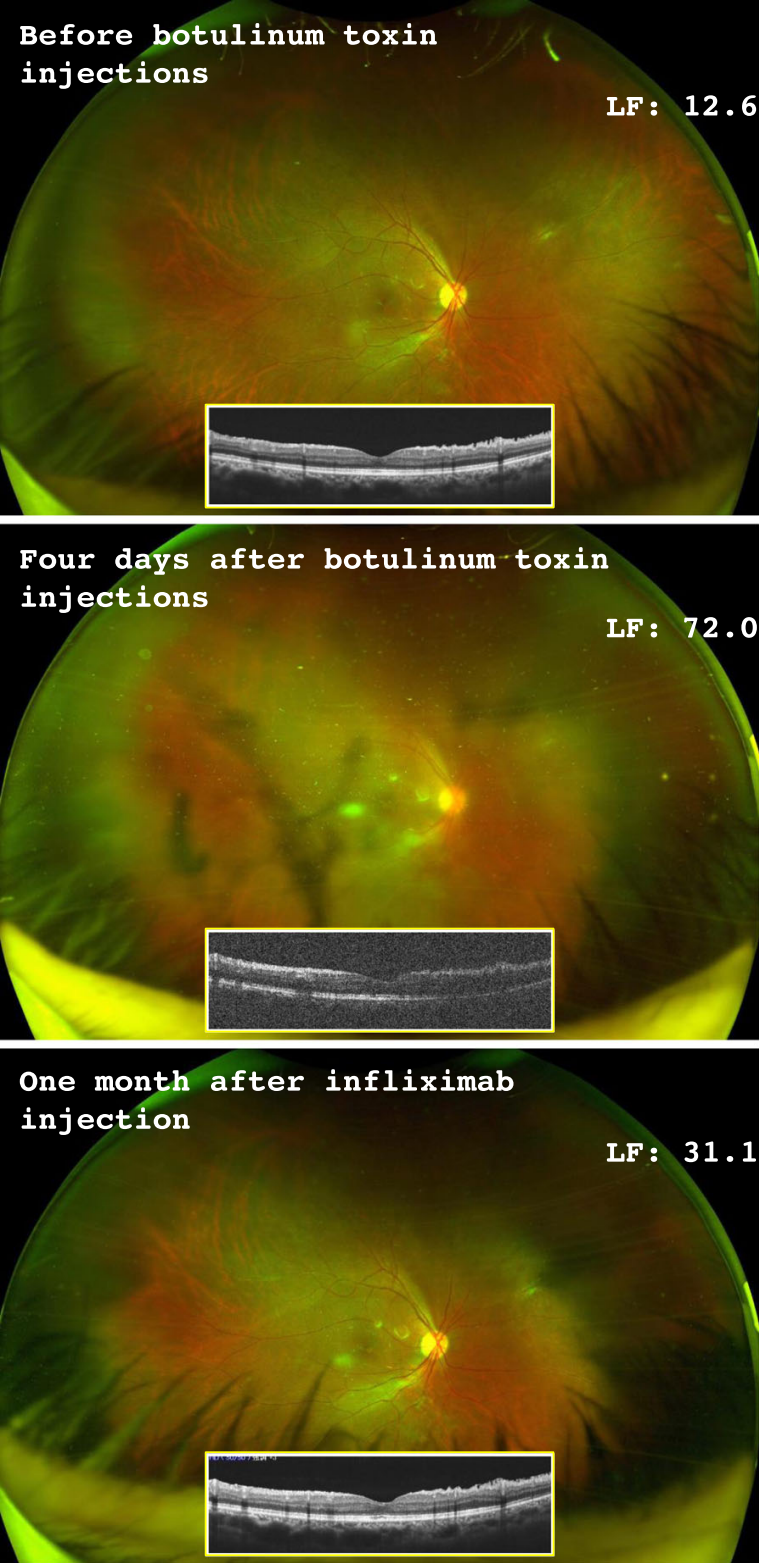
Ultra-widefield, high-resolution digital images, spectral domain optical coherence tomography (vertical section), and average aqueous flare values measured with laser flare meters for right eye. Before the botulinum toxin injections, the patient’s ocular condition was stable with no active uveitis at a routinely scheduled eye examination. Vitreous haze was graded as trace [7]. Two days later, he received botulinum toxin injections to treat axillary hyperhidrosis. Four days after botulinum toxin injections his right eye showed severe anterior vitreous opacity that was silhouetted against the retina, with elevated aqueous flare in anterior chamber. Vitreous haze was graded as 2+. No abnormal change was found in the retina. He received his regularly scheduled infliximab injection on the same day. One month after infliximab injection the right acute uveitis had attenuated considerably. Vitreous haze was graded as trace. Spectral domain optical coherence tomography did not detect macular edema after the acute uveitis, but severe vitreous opacity was clearly indicated by the cloudy image obtained 4 days after botulinum toxin injections. All the whitish lesions in the digital images are artifacts. *LF* aqueous flare value measured with laser flare meter in photon counts per millisecond (pc/ms)

A 53-year-old Japanese man visited our hospital for a scheduled follow-up examination for uveitis due to BD. His best corrected visual acuity (BCVA) was 20/16 in both eyes. Intraocular pressure was 15 and 11 mmHg in his right eye and left eye, respectively. Slit lamp biomicroscopy with Goldmann three-mirror examination did not reveal active uveitis in either eye. Vitreous haze was graded as trace [7]. Anterior chamber flare was evaluated based on the aqueous flare value measured with the Kowa FM-600® laser flare meter (Kowa Medicals, Nagoya, Japan) in photon counts per millisecond. Aqueous flare values measured with laser flare meter (LFs) were 12.6 and 7.6 photon counts per millisecond in his right eye and left eye, respectively; both were expected to be within normal range. An ultra-widefield (UWF™) high-resolution digital image (optomap®) was obtained with a Daytona imager (Optos, Scotland); no active uveitis lesions or vitreous opacity were detected (Fig. 1). Spectral domain optical coherence tomography (SDOCT; RS-3000, Nidek Co., Ltd, Aichi, Japan) was used to analyze the macular lesion; no macular edema was observed.

He had been diagnosed as having incomplete-type BD 11 years before. He was adequately treated with systemic corticosteroid and colchicine accompanied by occasional topical corticosteroids and mydriatics. Because his right visual function was disturbed by a preretinal membrane combined with vitreous opacity and secondary cataract in his right eye as a consequence of repeated bouts of severe uveitis, he underwent vitrectomy with inner limiting membrane peeling and phacoemulsification with intraocular implantation 2 years and 4 years after the diagnosis, respectively. Three years after the diagnosis he was given a systemic infusion of 5 mg/kg infliximab (Remicade®; Mitsubishi Tanabe Pharma Corporation, Osaka, Japan) every 8 weeks with no recurrence of uveitis attacks. He received no other treatments for BD besides the infliximab. He stated that his left eye had once shown mild intermediate to posterior uveitis, but that he had been completely stable since the commencement of systemic infliximab treatment.

Four days after the scheduled follow-up examination, he returned to our hospital to receive his regularly scheduled systemic infusion of infliximab. At the visit, he complained of blurred vision in his right eye. BCVA was 20/25 and 20/16 in his right eye and left eye, respectively. Intraocular pressure was 17 and 11 mmHg in his right eye and left eye, respectively. LF was 72.0 and 6.2 in his right eye and left eye, respectively. Slit lamp biomicroscopy with Goldmann three-mirror examination revealed severe AAU and deteriorated aqueous flare in the anterior chamber, with anterior vitreous opacity that was shown by the silhouette against the retina in his right eye (Fig. 1). Vitreous haze was graded as 2+. We did not observe any abnormal changes in his left eye (data not shown). SDOCT resulted in a cloudy image due to marked vitreous opacity, but we did not detect macular edema (Fig. 1).

He stated that 2 days prior he had received botulinum toxin (BOTOX®, botulinum toxin type A; GlaxoSmithKline K.K., Tokyo, Japan) 50 unit intracutaneous injections on both sides to treat axillary hyperhidrosis. Three hours after the injections, he noted severely increasing floaters and pain in his right eye. After he received the scheduled infliximab injection, however, he stated that the floaters and ocular pain in his right eye improved within a day. The severe vitreous opacity had attenuated considerably 1 month after the infliximab injection (Fig. 1). BCVA was 20/16 in both eyes. Intraocular pressure was 10 and 9 mmHg in his right eye and left eye, respectively. LF was 31.1 and 26.3 in his right eye and left eye, respectively. Vitreous haze was graded as trace. Finally, LF was 15.2 and 14.6 in his right eye and left eye 3 months after the infliximab injection, respectively.

After this event, he explained he had once received botulinum toxin injections in the same way last year. He received the scheduled infliximab injection 2 weeks prior. He felt no abnormality in both eyes, and a scheduled follow-up examination did not reveal active uveitis on that occasion.

## Discussion

In the present case, distinct botulinum toxin-induced AAU occurred only in the patient’s right eye. His left eye appeared to be normal except for a mild increase in LF 1 month after the AAU. Unlike his right eye, the patient’s left eye had not been severely damaged by uveitis associated with BD. This suggests that the botulinum toxin-induced AAU that occurred in the patient’s right eye may have been facilitated by a persistent disorder of the BAB as a consequence of BD and thus his left eye remained unaffected. Clinicians should consider the possibility that botulinum toxin could trigger AAU in eyes with damaged BABs as a consequence of lasting uveitis.

A high serum concentration of infliximab is expected to be maintained for a short period after infliximab administration, and it may be safer in terms of preventing uveitis in patients with BD. In the present event, our patient had received botulinum toxin injections more than 7 weeks after the last systemic infusion of infliximab. On the previous occasion, he fortunately received botulinum toxin injections just 2 weeks after infliximab administration, and thus we speculate he did not experience botulinum toxin-induced uveitis.

Konstantopoulou *et al*. evaluated the clinical grading of aqueous flare in uveitis according to the Standardization of Uveitis Nomenclature consensus, and compared the results with the readings of a laser flare meter (using the same laser flare meter system as we used in the present study) [8]. They reported that eyes graded as anterior chamber flare +2 had a median LF of 41.4 pc/ms, those graded as flare +1 had a median value of 18.4 pc/ms, and those graded as flare 0 had a median value of 9.9 pc/ms. According to these standards, 12.6 and 7.6 in the right eye and left eye, respectively, the aqueous flare values shown before the AAU, were equivalent to flare 0 although it may include individual variations, as described previously [8]. However, 72.0 in the right eye after the AAU was clearly abnormal and graded as anterior chamber flare more than +2.

Toll-like receptors (TLRs) are expressed in the uvea, macrophages, and peripheral blood monocytes. TLR4 is the primary signaling receptor for LPS-specific recognition and cellular activation [9]. Resident antigen-presenting cells in normal human uvea express TLR4 and its associated LPS receptor complex [10]. Chen *et al*. demonstrated that TLR4 expression increases in the iris and in macrophages in the iris tissue during EIU [11]. Primary cultures of human iris pigment epithelium cells express a functional LPS receptor complex and can promote ocular inflammation through secretion of several proinflammatory cytokines [12].

## Conclusions

We report an unusual case in which subcutaneous injections of botulinum toxin to treat axillary hyperhidrosis appeared to trigger AAU in a patient with BD even though he had not experienced a uveitis attack since starting periodic infliximab treatment 8 years ago. He received his regularly scheduled infliximab injection 2 days later, and the AAU resolved quickly with near-normal opacity, intraocular pressure, and LF a month later. Based on this temporal progress of uveitis, we concluded the AAU was not a simple relapse of BD but was induced by botulinum toxin injection, which was attenuated by infliximab, although the precise mechanism is still unclear. To the best of our knowledge, this is the first report of the triggering of AAU by botulinum toxin and effective attenuation of AAU by infliximab in a patient with BD.

## Abbreviations

AAU: Acute anterior uveitis
BAB: Blood–aqueous barrier
BCVA: Best corrected visual acuity
BD: Behçet’s disease
EIU: Endotoxin-induced uveitis
LF: Aqueous flare value measured with laser flare meter
LPS: Lipopolysaccharide
mRNA: messenger ribonucleic acid
SDOCT: Spectral domain optical coherence tomography
TLR: Toll-like receptor
TNF-α: Tumor necrosis factor alpha
